# Targeted Radionuclide Therapy for Patients with Metastatic Pheochromocytoma and Paraganglioma: From Low-Specific-Activity to High-Specific-Activity Iodine-131 Metaiodobenzylguanidine

**DOI:** 10.3390/cancers11071018

**Published:** 2019-07-20

**Authors:** Camilo Jimenez, William Erwin, Beth Chasen

**Affiliations:** 1 Department of Endocrine Neoplasia and Hormonal Disorders, The University of Texas MD Anderson Cancer Center, 1400 Pressler Street, Unit 1461, Houston, TX 77030, USA; 2 Department of Imaging Physics, The University of Texas MD Anderson Cancer Center, 1400 Pressler Street, Unit 1461, Houston, TX 77030, USA; 3 Department of Nuclear Medicine, The University of Texas MD Anderson Cancer Center, Houston, TX 77030, USA

**Keywords:** malignant pheochromocytoma and paraganglioma, norepinephrine transporter, low-specific-activity I-131-MIBG, high-specific-activity I-131-MIBG, clinical trials

## Abstract

Low-specific-activity iodine-131–radiolabeled metaiodobenzylguanidine (I-131-MIBG) was introduced last century as a potential systemic therapy for patients with malignant pheochromocytomas and paragangliomas. Collective information derived from mainly retrospective studies has suggested that 30–40% of patients with these tumors benefit from this treatment. A low index of radioactivity, lack of therapeutic standardization, and toxicity associated with intermediate to high activities (absorbed radiation doses) has prevented the implementation of I-131-MIBG’s in clinical practice. High-specific-activity, carrier-free I-131-MIBG has been developed over the past two decades as a novel therapy for patients with metastatic pheochromocytomas and paragangliomas that express the norepinephrine transporter. This drug allows for a high level of radioactivity, and as yet is not associated with cardiovascular toxicity. In a pivotal phase two clinical trial, more than 90% of patients achieved partial responses and disease stabilization with the improvement of hypertension. Furthermore, many patients exhibited long-term persistent antineoplastic effects. Currently, the high-specific-activity I-131-MIBG is the only approved therapy in the US for patients with metastatic pheochromocytomas and paragangliomas. This review will discuss the historical development of high-specific-activity I-131-MIBG, its benefits and adverse events, and future directions for clinical practice applicability and trial development.

## 1. Introduction

Pheochromocytomas and paragangliomas (PPGs) are neuroendocrine tumors originating in the paraganglia. The terms pheochromocytoma and paraganglioma apply to tumors originating in the adrenal medulla or the extra-adrenal paraganglia, respectively [1]. Because of their chromaffin cell origin, PPGs may synthesize and release catecholamines that predispose patients to cardiovascular and gastrointestinal disease [2,3,4,5]. In many patients, the clinical manifestations of PPGs are determined by the interactions of norepinephrine and/or epinephrine on the alpha- and beta-adrenergic receptors in multiple tissues and organs [4,5,6]. Thus, patients with PPGs may present with many and variable hormonal manifestations [6]. Interestingly, PPGs have a strong hereditary component, with more than 30% of patients carrying germline mutations in one of several susceptibility genes [7]. Fortunately, most patients with PPGs present with a localized disease that is curable with surgery [8]. However, 15–20% of patients with PPGs have metastatic disease [9]. Metastatic PPGs (MPPGs) are rare, with only 100–200 new cases diagnosed annually in the US [10]. Approximately 40–50% of MPPGs are associated with germline mutations of the succinate dehydrogenase subunit B (*SDHB*) gene [11]. However, MPPGs have several aspects that make them difficult to characterize. Unlike other cancers, PPGs have no histological, molecular, or biochemical markers to predict their malignant potential. Several scoring systems to predict the malignant potential have been proposed, e.g., the pheochromocytoma of the adrenal scaled score (PASS) and the age, size, extra-adrenal location, secretory type (ASES) systems [12,13]. PASS is unfortunately not reproducible and therefore no longer recommended for clinical practice [14]. ASES is not yet validated. Therefore, the definition of malignancy for these tumors relies on the presence of metastases [1]. Subsequently, a large majority of patients with MPPGs present with advanced disease. MPPGs mainly spread to the lymph nodes, skeletal tissue, lungs, and liver [15]. Only 50% to 60% of patients with MPPGs survive five years after their initial diagnosis [16]. Patients with MPPGs cannot be cured by surgery, and patients with progressive and/or symptomatic disease need systemic therapy. Patients who have asymptomatic disease with minimal or no progression do not require systemic therapy. Such patients, fortunately, are not uncommon. In fact, Hescot et al. calculated the MPPG progression-free survival rate at one year as 50% [17], and some of these patients may even have a normal life span [16,18].

I-131-MIBG was introduced in the 1980s as a potential systemic therapy for patients with progressive and/or symptomatic MPPGs that express the norepinephrine transporter (NET) in the tumor cell membranes [19]. At the beginning of the 21st century, a purified, high–specific-activity (HSA) I-131-MIBG was developed for the treatment of MPPG [20,21]. The results of a recently concluded pivotal phase 2 clinical trial of HSA-I-131-MIBG demonstrated that a substantial number of patients with MIBG-avid MPPGs benefit from this medication [22]. HSA-I-131-MIBG was approved by the US Food and Drug Administration (FDA) for the treatment of patients with MIBG-avid MPPGs in 2018. As the only FDA-approved therapy for patients with MPPGs, HSA-I-131-MIBG is considered the standard of care in the US for patients with MIBG-avid tumors. In this manuscript, we will discuss the development of I-131-MIBG as a treatment for patients with MPPGs, the NET as a target for I-131-MIBG therapy, the clinical benefits and indications of I-131-MIBG therapy, its toxicity, and clinical considerations that may improve its effectiveness.

## 2. I-131-MIBG

**Figure 1:**
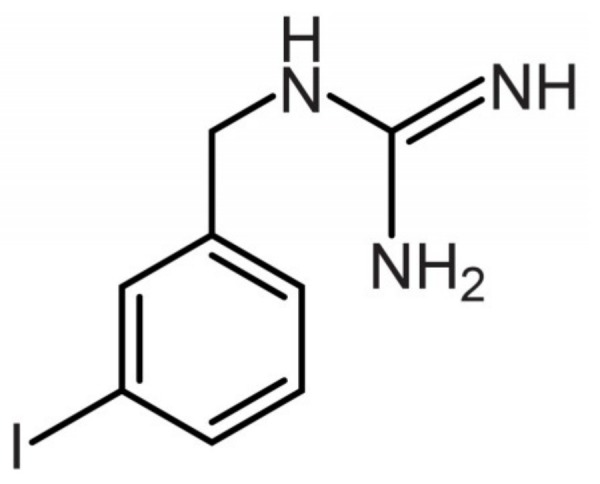
Molecular structure of MIBG.

MIBG was described by Wieland et al. in 1979 at the University of Michigan [23,24]. MIBG contains a benzyl and a guanidine group [24]. MIBG is a substrate of the NET. However, unlike norepinephrine, MIBG has little or no affinity for adrenergic receptors [23]. MIBG is characterized by iodination in the meta position of the benzyl ring; this iodination makes MIBG a very stable molecule that is highly resistant to in vivo metabolism (Figure 1).

MIBG is eliminated mainly through the kidneys, and approximately 90% of the MIBG is excreted intact in the urine. Forty to fifty percent of the urinary excretion happens within 24 h, and 70% to 90% of MIBG is recovered in urine within 96 h [25].

MIBG can be radiolabeled with I-123 or I-131 [26]. Like norepinephrine, radiolabeled MIBG is captured by the NET. Both radioisotopes emit gamma radiation that can be captured by a scintillation (or gamma) camera (i.e., scintigraphy). I-123 has a shorter half-life than I-131. However, I-123 offers better imaging characteristics than I-131 with conventional gamma cameras and is the preferred radioisotope for diagnostic purposes. The long half-life of I-131 makes it possible to obtain delayed images with this radioisotope and allows a longer duration of radioactivity concentration in PPGs [27]. Diagnostic imaging with either I-123- or I-131-radiolabeled MIBG is used to stage PPG and to detect occult tumors in patients with adrenergic symptoms and elevated catecholamine levels. In fact, I-131-MIBG was approved in 1994 by the FDA as an imaging agent for the localization of neuroblastomas and PPGs. For diagnostic purposes, I-123-MIBG and I-131-MIBG are provided at a dose of 5 mCi (185 MBq).

At a high dose level, I-131-MIBG may emit enough beta (primarily) and gamma (secondarily) radiation to cause lethal cellular damage. Therefore, patients with MPPG, neuroblastoma, and other MIBG-avid tumors treated with a high dose of I-131-MIBG may exhibit clinical benefits [28]. Therapeutic activities may reach 1000 mCi or even more when given sequentially (i.e., over multiple treatments). I-131 decays with a half-life of 8.03 days to the very stable xenon-131. During the decay process, one or more gamma and x-Ray photons and a negative beta particle are emitted. The primary photon (gamma emission) has an energy of 364.5 keV, and the predominant beta emission has average and maximum energies of 191.5 keV and 606.3 keV, respectively. The gamma photon is therefore highly penetrating owing to its relatively high energy. Because body tissues poorly attenuate the gamma photons, about 50% of these photons are still present approximately 6 cm from the site of decay [29]. In contrast, the beta particle has an electron mass and a negative electrical charge (−1) and interacts with matter very near the source of decay. Indeed, the I-131 beta particles have quite limited penetration, and their radiation effects concentrate near the decay site (0.4 mm average, 2.3 mm maximum path lengths). Energy is transferred along the beta particle path as the particle slows. The linear energy transfer of the beta particles is much higher than that of the gamma photons. The release of energy from the beta particles results in ionization and the formation of free radicals that damage DNA (via single- or double-strand brakes) and general cellular physiology. I-131-MIBG may therefore control one of the most important hallmarks of cancer, sustained replicative signaling [30].

## 3. The Norepinephrine Transporter

**Figure 2:**
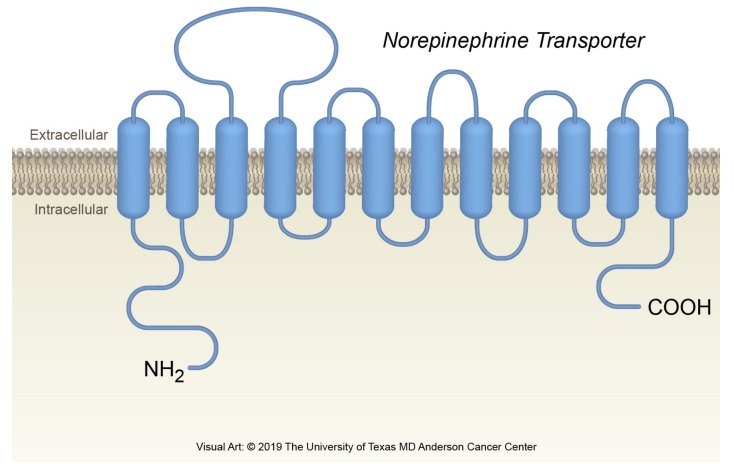
The norepinephrine transporter.

Norepinephrine is a monoamine that works as a neurotransmitter in the central and autonomic nervous systems. In the sympathetic autonomic nervous system, most postganglionic sympathetic neurons lead from the sympathetic chain to the target visceral organ where they end with bulbous enlargements, or varicosities [31,32]. These varicosities release norepinephrine into their surrounding environment [33], and norepinephrine’s interactions with the alpha- and beta receptors mediate the stress- and fear-related responses of the autonomic nervous system [32,34]. The concentrations of norepinephrine in the surroundings are regulated by the NET [35]. The NET is a vesicular monoamine transporter and a member of the family of solute carrier 6 transporters [36]. The NET is embedded in the cell membrane of the postganglionic neuron—it is not located in the cell membranes of the visceral organs. The NET structure has 12 alpha-helical transmembrane-spanning domains interconnected with flexible intra- and extracellular loops (Figure 2).

**Figure 3:**
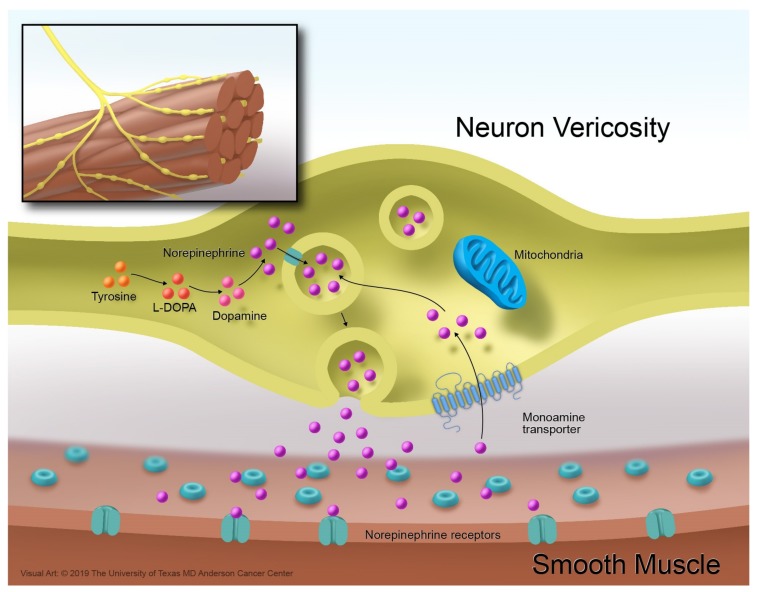
Norepinephrine reuptake mechanism.

The NET terminates the action of norepinephrine by translocating this monoamine from its surroundings inside the postganglionic neuron. Once in the cytoplasm, the norepinephrine is stored in vesicles (Figure 3) [37].

Because of their origin, several neuroendocrine tumors express the NET on their cell membranes. These tumors include gastroenteropancreatic neuroendocrine tumors, medullary thyroid carcinomas, and PPGs [38,39,40]. Approximately 50–60% of PPGs express the NET in their cell membranes [41,42]. It is not known why some PPGs do not express the NET in the cell membrane. In fact, the NET can be expressed by hormonally and non-hormonally active, adrenal and extra-adrenal, and metastatic and nonmetastatic PPGs [40]. Some small case series have indicated that PPGs that are associated with pseudohypoxia may have lower or no expression of the NET compared with other tumors [43]. However, clinical experience and observations derived from phase 2 clinical trials for patients with MPPGs treated with radiolabeled MIBG indicate that patients with *SDHB* mutations may respond very well to therapy [44]. Interestingly, one study suggests that the expression of the NET does not correlate with the degree of MIBG uptake [43], and some studies hypothesize that MIBG uptake may also depend on the presence of catecholamine-releasing granules [45].

## 4. Classification of MIBG Therapy Based on Manufacturing Aspects

MIBG can be classified as conventional low specific activity (LSA) or HSA. Both LSA-I-131-MIBG and HSA-I-131-MIBG are radiolabeled with I-131 at the meta position of the benzyl ring and exhibit the same structure [46]. However, their manufacturing processes differ. LSA-I-131-MIBG is produced by isotope exchange. Isotopic exchange yields in a considerable amount of cold carrier MIBG in the final solution. In fact, the ratio of radioactive MIBG to nonradioactive MIBG molecules is about 1:2000 at a given level of radioactivity [46]. This issue has therapeutic implications. Non-radiolabeled MIBG competes with radiolabeled MIBG for the NET and therefore may prevent the delivery of lethal radiation to the target cells, making the treatment with LSA-I-131-MIBG less effective. Furthermore, the saturation of the NET by cold molecules of MIBG prevents the reuptake of norepinephrine, increasing norepinephrine’s extracellular concentrations and subsequent interactions with the alpha- and beta-adrenergic receptors, and thereby exacerbating the manifestations of catecholamine excess [44].

Unlike LSA-I-131-MIBG, HSA-I-131-MIBG is produced by an Ultratrace methodology, or electrophilic iododesilylation [46]. This methodology constitutes a substantial improvement in the manufacturing of radiolabeled MIBG and prevents unlabeled or cold MIBG from being carried through from the production reaction to the final product. The final product is therefore highly specific, no-carrier-added I-131-MIBG. HSA-I-131-MIBG results in an increased cellular uptake of lethal radioactivity, approximately 100–200 times higher than that of LSA-I-131-MIBG [46]. Compared with LSA-I-131-MIBG, HSA-I-131-MIBG is less likely to saturate the NET and subsequently may cause fewer hormonal complications [46]. Table 1 compares LSA- and HSA-I-131-MIBG.

## 5. Patient Preparation

Patient time commitment and preparation, as well as potential side effects, are similar if one were to consider HSA-I-131 or LSA-I-131-MIBG therapy. Appropriate instruction is a vital component for successful therapy, and the clear communication of the timelines, preparatory steps, and side effects/risks of treatment is crucial. For the dosimetry phase, patients need to be aware of the three separate visits to the nuclear medicine department (day of infusion with subsequent imaging, 1–2-day imaging, and 2–5-day imaging). The dosimetry imaging is comprised of planar whole-body scans, each of which take approximately 30–45 min. Treatment occurs between 7 and 14 days following the dosimetric evaluation; the time gap allows for the manufacture and shipment of the treatment activity. This therapy requires hospital admission, and patients should plan for an inpatient stay of up to seven days for radiation safety precautions.

Patient preparation includes screening for the use of medications that may interfere with dosimetry calculations or treatment efficacy [48]. Drugs that reduce catecholamine uptake or deplete catecholamine reserves should be discontinued for at least five half-lives prior to dosimetry through at least seven days after therapy. The medication list is rather lengthy and includes certain blood pressure medications, antidepressants, tramadol, and decongestants (pseudoephedrine). Of note, contraindicated blood pressure medications include the combined alpha/beta blocker labetalol and calcium channel blockers. In addition, some over the counter botanicals such as St John’s Wort and yohimbine are also contraindicated. Patients should stay well hydrated during this therapy with fluid intake of at least 2 L per day beginning at least one day before and continuing at least one week after treatment. Given that the primary elimination route of therapy is via urinary excretion, adequate hydration will reduce radiation dose to the nontargeted organs. Because of the potential nontargeted uptake of I-131-MIBG within the thyroid gland, patients receive a thyroid blocking agent (potassium iodide) beginning one day before the treatment administration and continuing for 10 days following dosing [48].

There are potential short-term side effects of I-131-MIBG therapy that should be described to patients. The use of premedications to prevent infusion-related nausea and vomiting is advised. Episodes of increasing hypertension can occur within 24 h of treatment administration. Frequent blood pressure monitoring is suggested and should occur routinely as part of the hospitalization for the therapy. Medications for blood pressure control can be prescribed and administered as needed during the hospital stay. Because of the risk of further myelosuppression, minimum baseline laboratory values for platelet count and absolute neutrophil count (ANC) have been established at 80,000 µL and 1200 µL, respectively. Typically, the second treatment is scheduled for 90 days following initial therapy but should be delayed until platelets and neutrophils return to baseline or the normal range [49]. Second treatment dose reduction is recommended in the settings of measured platelet count less than 25,000 µL or less than 50,000 µL with active bleeding, ANC less than 500/µL or febrile neutropenia, and life-threatening anemia (hemoglobin less than 6.5 g/dL) for more than seven days.

Patients should also be educated about the possible long-term side effects of treatment. Cumulative radiation exposure contributes to an increased risk of cancer, and, although rare, secondary myelodysplastic syndrome and acute leukemia have occurred with this therapy [22]. All radiopharmaceuticals have the potential to cause harm to a fetus, and pregnancy testing should be performed prior to therapy for all patients with child-bearing potential. It is recommended that females of reproductive potential use contraception during therapy and for seven months after the final dose and males with female partners of reproductive potential use contraception during treatment and for four months after the last dose. In addition, the degree of radiation exposure to the testes and ovaries is within range to expect potential temporary or permanent infertility. Because of the risk of infertility, a discussion should be held with applicable patients regarding the option of egg or sperm banking. Breastfeeding is contraindicated during treatment with I-131-MIBG. Hypothyroidism is a risk even with adequate thyroid blocking. Renal toxicity is also a possibility despite recommended hydration. Of note, I-131-MIBG has not been studied in patients with severe renal impairment. Similarly, patients with evidence of liver dysfunction, a history of liver disease, and certain prior treatments with radiotherapy have not been assessed.

## 6. LSA-I-131-MIBG for the Treatment of Patients with MPPG

The first report of the use of radiolabeled MIBG as a potential therapy for patients with MPPG was published in 1984 [50]. Since then, several cases and retrospective studies and 1 prospective phase 2 clinical trial with LSA-I-131-MIBG have been reported. These publications suggest that LSA-1-131-MIBG could be an effective treatment for some patients with MPPG. Nevertheless, only a few of these publications allow conclusions to be made about how MIBG could be prescribed to such patients and which benefits might be expected. The highlights of the most relevant studies are presented below. These studies had a clear description of the intervention, evaluated and defined tumor responses according to established radiographic criteria, and included an acceptable number of patients (*n* ≥ 10). The studies are classified by treatment dosage as follows: low dose (80–200 mCi/person/session), intermediate dose (201–500 mCi/person/session), and high dose (>500 mCi/person/session).

### 6.1. Retrospective Studies with Low Doses of LSA-I-131-MIBG

The first clinically relevant study was a review of retrospective studies of patients with MPPGs treated with LSA-I-131-MIBG from 1983 to 1997 [51,52,53,54,55,56]. Although these studies came from 24 centers in 10 countries and had different methodologies [51], the analysis showed that sequential low doses of LSA-I-131-MIBG could have a positive impact on the clinical outcomes of some patients with MIBG-avid MPPGs [51]. The study also confirmed that patients with MIBG-avid tumors (as demonstrated by pretreatment MIBG scanning), but not patients with tumors that are not MIBG avid, may benefit from LSA-I-131-MIBG [51]. The study included 116 patients who received a mean treatment dose of 158 mCi (96–300 mCi), a mean of 3.3 repeated treatments, and a mean cumulative dose of 490 mCi (96–2322 mCi) of LSA-I-131-MIBG [51]. After treatment with LSA-I-131-MIBG, 30% of patients exhibited an objective tumor response, including 4% of patients with tumor disappearance or complete responses (CRs) and 26% of patients with partial responses (PRs). Furthermore, 57% of patients had stable disease (SD). Only 13% of patients did not respond and had progressive disease (PD). The patients with CRs were followed up for 16–58 months and had no evidence of recurrence at the time of publication [51]. Biomarker responses were also evaluated and included measurements of plasma and/or urinary catecholamines. Biomarker responses defined as CRs or PRs were noted in 45% of patients. Catecholamine levels normalized in 13% of patients, 32% had partial improvements, and 45% showed no changes. Of interest, 76% of patients with adrenergic manifestations had symptomatic improvement [51]. This study also suggested that LSA-I-131-MIBG may improve overall survival (OS). Patients whose disease responded to LSA-I-131-MIBG had a 33% mortality rate at a median 22 months after therapy, whereas those whose disease did not respond had a 45% mortality rate at a median 13 months.

Less heterogeneous retrospective studies from referral centers have also suggested that low doses of LSA-I-131-MIBG (median <200 mCi) may bring clinical benefits and minimal toxicity for patients with MPPGs. Gedik et al. retrospectively reviewed the outcomes of 19 patients with MIBG-avid tumors treated with a median initial dose of 200 mCi and a median cumulative dose of 600 mCi of LSA-I-131-MIBG [57]. Of 17 evaluable patients, 47% had radiographic responses according to World Health Organization (WHO) criteria and 67% had biochemical responses. A symptomatic response was noted in 89%. The study described a progression-free survival (PFS) of 24 months and an OS of 42 months. There were no CRs [57].

Shilkrut et al. described 10 patients with MPPGs treated with a mean initial dose of 145 mCi of LSA-I-131-MIBG. The median total administered dose was 310 mCi (100–605) [58]. The best radiographic responses according to response evaluation criteria in solid tumors (RECIST) 1.0 were PR and SD in 30% and 50% of patients, respectively. There were no CRs, and 20% of patients did not respond to therapy [58].

Wakabayashi et al. published the largest single-institution retrospective study of single low doses of LSA-I-131-MIBG for the treatment of MPPG [59]. This study included 26 patients treated with a median dose of 200 mCi. Of 20 patients evaluable for radiographic responses, none achieved a PR or CR, 85% had SD as the best radiographic response, and 80% died during the follow-up period. The results of this study suggested that small, single doses of LSA-I-131-MIBG have a limited antineoplastic benefit.

The retrospective studies discussed above indicated that the major toxicity associated with small doses of LSA-I-131-MIBG was a reversible bone marrow insufficiency. Approximately 20–30% of patients experienced transient grade 3 or 4 anemia, thrombocytopenia, and/or leucopenia. A few patients had a mild exacerbation of symptoms of catecholamine excess [57,58,59].

### 6.2. Retrospective Studies with Intermediate Doses of LSA-I-131-MIBG

Safford et al. described 33 patients treated with a mean initial dose of 388 mCi and mean cumulative dose of 550 mCi (70-1223 mCi) [60]. The study showed an objective tumor response rate (ORR) of 38%, a biomarker response rate of 60% (urine catecholamines and metanephrines), and improvement of clinical manifestations in 86% of patients. Eight patients achieved a CR according to WHO criteria [60]. Four patients had transient bone marrow insufficiency. Patients whose disease responded to LSA-I-131-MIBG had significantly longer OS when compared with those whose disease did not (4.7 versus 1.8 years, *p* < 0.01). Of interest, patients treated with initial doses of 500 mCi had a survival advantage when compared with individuals treated with doses lower than 500 mCi (3.8 versus 2.6 years, *p* = 0.02) [60].

Castellani et al. evaluated 24 patients with MPPGs treated with LSA-I-131-MIBG [61]. The study included 2 historical cohorts. One group included 10 patients treated with a fixed dose of 150 mCi per session, and a second group included 14 patients treated with 250–350 mCi per session. The median cumulative dose was 1065 mCi (148–1800 mCi) for the low-dose group and 651 mCi (250–1546 mCi) for the intermediate-dose group. ORRs were similar for both groups, with 10% to 15% of patients exhibiting a CR and 20–23% having PRs. Patients treated with intermediate doses achieved radiographic responses faster than patients treated with low doses. Patients treated with intermediate doses also exhibited more frequent and severe bone marrow suppression. However, no patients developed permanent bone marrow insufficiency.

### 6.3. Prospective Clinical Trial with High-Dose LSA-I-131-MIBG

A phase 2 study of high-dose LSA-I-131-MIBG for patients with MPPGs included 50 patients treated with a mean initial dose of 800 mCi (492–1160 mCi) and a median cumulative dose of 1100 mCi (492–3191 mCi) [44]. The overall response rate was 22% and included 14% of patients with PR and 8% with CR. The ORR was 57% and included these plus 35% of patients who achieved some degree of regression (<30% of tumor size reduction as per RECIST criteria). Another 8% of patients had no changes in tumor size at 1 year. However, 35% of patients did not respond to treatment and had disease progression within 1 year of LSA-I-131-MIBG therapy [44]. All patients with MPPGs and germline *SDHB* mutations exhibited clinical benefits [44]. The study did not report whether the other patients were tested for susceptibility genes; therefore, it was not possible to determine if *SDHB* status could predict OS. The 5-year OS rate was 64%, and the 5-year event-free survival rate was 47% [44]. More than 80% of patients experienced grade 3–4 bone marrow toxic effects. Four patients experienced prolonged bone marrow suppression that required autologous stem cell rescue. Two patients developed lethal myelodysplasia after two and three treatments with high doses of LSA-I-131-MIBG. Of interest, 10 patients had hypertension during the infusion of LSA-I-131-MIB, including seven patients who had severe (grade 3) hypertension [44].

Other serious adverse events included two cases of acute respiratory distress syndrome (ARDS), which occurred within six months after infusion, and two cases of bronchiolitis obliterans organizing pneumonia. Two of these patients with respiratory complications had baseline nephrotic syndrome [44]. No previous studies had described respiratory adverse events associated with MIBG therapy, but a subsequent study indicated that the risk of ARDS was strongly associated with preexisting proteinuria regardless of the dose of LSA-1-131-MIBG, number of treatments, age, catecholamine levels, and severity of hypertension [62]. Therefore, urine protein excretion should be evaluated before high-dose LSA-I-131-MIBG therapy [62,63].

### 6.4. Remarks on the Retrospective and Prospective Studies with LSA-I-131-MIBG

The implementation of LSA-I-131-MIBG therapy has lacked an evidence-based standard development, and consequently the regimens vary among hospitals and treatment teams. In addition, the rarity of MPPG and the fact that in many circumstances LSA-I-131-MIBG is the only available therapeutic option (i.e., patients/clinicians frequently decline chemotherapy) have made prospective and systematic study of this medication difficult. A retrospective meta-analysis and systematic review of 17 studies that included 243 patients with MPPGs treated with LSA-I-131-MIBG indicated that some patients may have benefited from this therapy [64]. These patients showed antineoplastic effects and symptomatic improvement; tumor stabilization and a substantial reduction of the catecholamine surge were the most common positive outcomes [64]. CRs were exceedingly rare [64]. The evaluation of antineoplastic effects had several limitations. Radiographic assessments were not always standardized, and only four studies used RECIST criteria to assess tumor responses [44,58,64,65,66]. Furthermore, only one study required that patients had PD before treatment with LSA-I-131-MIBG was provided [61]. Therefore, the lack of tumor progression that was observed in some patients after treatment may not be related to the antineoplastic effects of LSA-I-131-MIBG. Hescot et al. described the natural course of treatment-naïve patients with MPPGs [17]. These patients may indeed exhibit heterogeneous outcomes, including indolent disease. Approximately 50% of patients with MPPGs may have minimal or no progression of the disease over a period of one year or longer [17]. Subsequently, the reported PFS and OS rates described by the studies with LSA-I-131-MIBG are not interpretable [49,64]. Although some studies suggest that patients with MPPGs treated with LSA-I-131-MIBG had an improvement of their clinical manifestations [51,60], none of these studies used standardized instruments to evaluate quality of life. Some studies have suggested that a better ORR and a longer OS could be achieved when prescribing high individual doses of around 800 mCi or 11.5 mCi/kg [44]. However, high doses of LSA-I-131-MIBG were associated with a higher risk of severe systemic toxic effects [44]. In fact, severe cardiovascular and pulmonary adverse events were mainly seen in patients treated with a high dose rather than a low or intermediate dose of LSA-I-131-MIBG [20]. Furthermore, no studies performed individual tumor dosimetry to optimize dose delivery. Together, all these issues point to a remarkable limitation in the recognition of an efficient dose of LSA-I-131-MIBG: the lack of evidence derived from a phase one clinical trial in patients with MPPGs.

## 7. HSA-I-131-MIBG for the Treatment of Patients with MPPG

Unlike LSA-I-131-MIBG, HSA-I-131-MIBG has followed the standard procedures for drug discovery and development, starting with preclinical studies and then moving into sequential phase one and two clinical trial evaluations [20,22,67,68,69,70]. Owing to the rarity of MPPG and the lack of a standard therapy, a phase three clinical trial was considered very difficult to pursue.

### 7.1. Phase 1 Clinical Trials with HSA-I-131-MIBG

An initial phase one study investigated the pharmacokinetics, dosimetry, and safety of HSA-I-131-MIBG in 11 patients with MPPGs or gastroenteropancreatic neuroendocrine tumors [20]. This study employed only 5 mCi of HSA-I-131-MIBG. These patients received a supplementary 185 µg of unlabeled MIBG to simulate a therapeutic dose. There were no side effects attributable to this intervention [20]. Approximately 80% of the medication was excreted unaltered in the urine over a period no longer than 120 h [20]. This finding was similar to those of an organ dosimetry study showing that organ distribution and whole-body retention of HSA-I-123-MIBG in cancer-free individuals were similar to that of LSA-I-123-MIBG [70].

An open label, multicenter, dose-escalation, phase 1 clinical trial for 21 patients with metastatic and/or unresectable PPGs was designed to determine the maximum tolerated dose of HSA-I-131-MIBG [69]. This study evaluated side effects, ORR, biomarker response (urine metanephrines and serum chromogranin A levels), and estimated radiation absorbed doses in target lesions and organs [69]. Patients were treated with HSA-I-131-MIBG through sequential dose-escalation cohorts. Titration began with three patients at 6 mCi/kg and proceeded according to a standard modified Fibonacci 3 + 3 trial design, with dose increases at 1-mCi/kg increments until the maximum tolerated dose was established. The dose-limiting toxicity (DLT) was bone marrow insufficiency [69]. Four patients experienced DLTs: two developed neutropenia, one had thrombocytopenia, and one had a combination of febrile neutropenia and thrombocytopenia. All 4 patients recovered from the DLTs within one month [69]. The patients who developed DLTs were treated with an HSA-I-131-MIBG dose higher than 500 mCi (524–680) or the equivalent of 9 mCi/kg [69]. Of interest, this phase 1 clinical trial did not report serious cardiovascular adverse events [69]. In fact, one-third of patients exhibited a better blood pressure control. Furthermore, four patients achieved PRs. The maximum tolerated dose was determined as 8 mCi/kg. Based on these results, a dose of 500 mCi was selected to develop a phase two clinical trial [69].

### 7.2. Phase 2 Clinical Trial

The efficacy of HSA-I-131-MIBG was evaluated through a pivotal multicenter, open-label phase two clinical trial for patients with metastatic, recurrent, and/or unresectable PPGs [22]. Because MPPG patients have substantial symptom burden from catecholamine excess and tumor burden from disease progression, the clinical trial endpoints were selected to specifically address these dual burdens. The FDA recommended blood pressure control as the primary objective of this trial. This recommendation was based on the fact that blood pressure control is a measurable clinical endpoint that impacts cardiovascular mortality, as demonstrated by several clinical trials that have evaluated treatments for patients with hypertension [71]. Of interest, no previous studies of LSA-I-131-MIBG considered blood pressure control as the primary endpoint. Blood pressure control rate was defined as the number of patients able to have a discontinuation or a reduction from baseline use of antihypertensive medications (number of antihypertensives or dose) by at least 50% for at least 6 months. An important secondary endpoint was the ORR per RECIST 1.0. Patients received a dosimetric dose (3–6 mCi) and up to two therapeutic doses (8 mCi/kg) up to a maximum of 500 mCi of HSA-I-131-MIBG per dose. The two doses were given approximately three months apart. Sixty-eight patients received at least one dose, and 50 received a second dose.

Seventeen patients (25%) achieved the primary endpoint; 16 of these received two doses of HSA-I-131-MIBG. The median duration of clinical benefit in the 17 patients who met the primary endpoint was 13.3 months. Of interest, 33 of the 68 patients enrolled in the trial had a reduction in the dose of antihypertensive medications by at least 50% for any duration [22].

**Figure 4:**
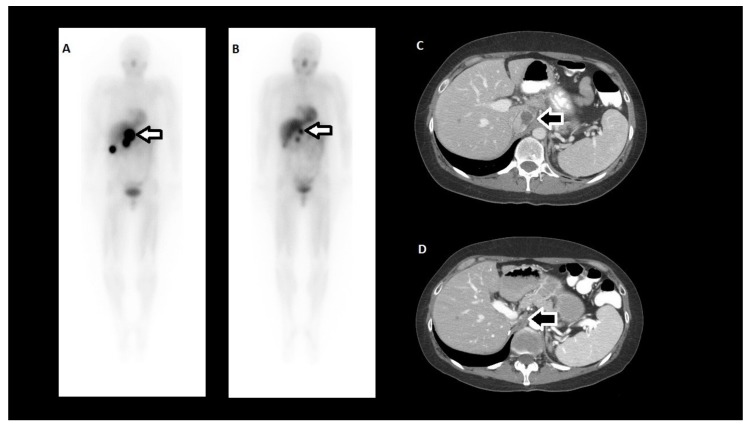
Clinical example of a tumor radiographic response to HSA-I-131-MIBG in a patient treated at The University of Texas MD Anderson Cancer Center. (**A**) HSA-131-MIBG scan 3 days after the first treatment; (**B**) HSA-131-MIBG scan 3 days after the second treatment; (**C**) Computed tomography prior to the first HSA-131-MIBG treatment; (**D**) Computed tomography 11 months after the second HSA-131-MIBG treatment (44% reduction in longest tumor dimension). Arrows indicate the tumor.

Overall, 92.2% of patients achieved a confirmed PR or SD as the best tumor response, but there were no CRs [22]. PRs were only noted in patients treated with two doses of HSA-I-131-MIBG. In fact, 30% of patients treated with two doses exhibited a PR, and 68% had SD. Most patients (71.4%) treated with a single dose of HSA-I-131-MIBG had SD as the best tumor response. All patients who met the primary endpoint achieved either a PR or SD. Of interest, 31 patients who did not achieve the primary endpoint exhibited some degree of tumor regression, including 12 patients who had a PR. Of the 45 patients available for evaluation 12 months after initial treatment, 16 patients had PR (36%), 24 had SD (53%), and five had PD (11%). The number of PRs increased over time, an observation that suggests that HSA-I-131-MIBG has persistent antitumor effects (Figure 4). Plasma and urinary catecholamine and metanephrine levels and serum chromogranin A concentrations correlated with the clinical and radiographic responses [72].

The most common (≥50%) treatment-emergent adverse events in all patients who received any dose of HSA-I-131-MIBG were nausea, fatigue, and myelosuppression [22]. Constitutional adverse events were mild and self-limited. Bone marrow suppression happened in more than 50% of patients. The most common bone marrow deficiency was thrombocytopenia (66.2%), followed by leucopenia (55%), and anemia (54%). Bone marrow suppression reached a nadir 4–8 weeks after treatment infusion. Supportive measures such as platelet and/or red blood cell transfusion and/or the use of granulocyte colony stimulating factors were required in 23% of patients. All patients recovered from bone marrow insufficiency, and stem cell transplantation was not required. More than two years after treatment, one patient developed myelodysplasia and one patient developed acute myelogenous leukemia. Of interest, no severe hypertension or catecholamine crises were noted during or immediately after the HSA-I-131-MIBG infusion [22].

### 7.3. Biodistribution, Dosimetry, and Therapeutic Administration

The phase two clinical trial included a dosimetric phase in which the biodistribution of HSA-I-131-MIBG and radiation absorbed doses in the primary organs at risk were estimated on the basis of scintigraphic imaging of a tracer administration of HSA-I-131-MIBG [20,73] (The FDA-approved therapeutic regimen requires this same pretreatment dosimetric phase [22]). A 1-mCi/mL saline (0.9% sodium chloride) solution of HSA-I-131-MIBG, nominally 6 mCi total dose in a 10-mL syringe, was infused intravenously over 1 min. Whole-body planar images were then acquired immediately and again at 1–2 days and 2–5 days.

The lung, liver, and kidney radiation absorbed doses were corrected for the patient’s organ masses (computed tomography scan volume estimates multiplied by 1.03 g/mL density for liver and kidney and 0.25 g/mL for lung). Finally, per-treatment prescribed dose was either 8 mCi/kg (but not more than 500 mCi) or that which would result in lung, liver, and kidney estimated doses that would not exceed 17.5 Gy, 30 Gy, and 23 Gy, respectively. The estimated cumulative total body radiation absorbed dose for a subset of the expanded access program patients at our institution (*n* = 7), who received a cumulative activity of 981 ± 107 mCi (range: 789–1049 mCi), was 3.10 ± 0.57 Gy (range: 2.40–3.97 Gy). The average normalized total body dose was 0.0034 Gy/mCi, close to the published value for the phase 1 clinical trial (0.0037 Gy/mCi) [20]. (The overall dosimetry results for the phase 2 and EAP clinical trials of HSA-I-131-MIBG have not yet been published.)

Due to the very high therapeutic amount of I-131 radioactivity, the HSA-I-131-MIBG treatment required the patient to be admitted to the hospital and remain in radiation isolation until he or she could be released to the public according to local radiation control regulations. The prescribed therapeutic dose was delivered in a 50-mL vial containing 13.5 to 16.5 mCi/mL HSA-I-131-MIBG and administered intravenously via an infusion pump at a rate of 100 mL/h. Prior to the start of actual dose administration, saline was infused for a minimum of 15 min at 200 mL/h to ensure that the intravenous access was operational and to prime the infusion line. Following completion of the HSA-I-131-MIBG dose administration, the infusion line was flushed with an additional 50 mL of saline at 200 mL/h to ensure the patient received the entire dose of HSA-I-131-MIBG.

### 7.4. LSA-I-131-MIBG vs. HSA-I-131-MIBG in the US

No clinical trial has compared LSA-I-131-MIBG with HSA-I-131-MIBG. Therefore, it is not possible to indicate which treatment is more effective. However, unlike LSA-I-131-MIBG, HSA-I-131-MIBG was approved in 2018 by the FDA. The approval was based on the findings of the phase 2 trial that included better blood pressure control, a disease control rate of 92.2% at one year, and acceptable toxicity [22] and its much higher levels of radioactivity delivered to the tumor per dose when compared with LSA-I-131-MIBG. For many years, health insurance companies and federal health programs such as Medicare and Medicaid declined to cover treatment with LSA-I-131-MIBG because it did not have an approved indication by the FDA. As a result, a number of patients could not receive treatment with LSA-I-131-MIBG. The FDA’s approval of HSA-I-131-MIBG is an important milestone in clinical practice because now patients with MIBG-avid MPPG have access to treatment that is most likely covered by their public or private health insurance. As HSA-I-131-MIBG is by definition the standard of care for the treatment of patients with MIBG-avid MPPG, any future clinical trials in the US will most likely evaluate HSA-I-131-MIBG rather than LSA-I-131-MIBG.

## 8. Future Directions

### 8.1. Surgery and I-131-MIBG

Patients with MPPGs frequently have primary tumors that are larger than 5 cm in association with extensive synchronous metastases [9]. Polyostotic disease, bilobar liver infiltration, multiple lung nodules, and large conglomerates of lymphadenopathy are not uncommon [16,49]. In patients with such conditions, surgical resection of the primary tumor has been associated with clinical benefits [74,75]. Hormonal manifestations may improve after surgery [74,75], patients seem to have a lower rate of anatomical complications (e.g., urinary or gastrointestinal tract obstruction) [74], and their OS rates seem longer [74]. Furthermore, the removal of the primary tumor may bring an opportunity to enhance the concentration of I-131-MIBG in metastatic sites. This phenomenon has been demonstrated in patients with thyroid cancer of follicular origin, in whom a total thyroidectomy with cervical neck dissection before iodine ablation therapy increased the efficacy of radiolabeled iodine [76]. Therefore, the surgical resection of the primary tumor and/or metastasectomy, if possible, should be considered in preparation for therapy with I-131-MIBG.

### 8.2. Chemotherapy and I-131-MIBG

Approximately 30–40% of patients with progressive MPPG respond to chemotherapy with cyclophosphamide, vincristine, and dacarbazine (CVD) [77]. Responders most often achieve a PR or SD and may experience a longer survival [77,78]. Patients with rapidly progressive MPPG are better candidates for chemotherapy than radiolabeled MIBG as the latter works slowly [16,49,63]. Like radiolabeled MIBG, CVD may cause bone marrow insufficiency and (rarely) predispose patients to secondary hematological malignancies (e.g., myelodysplasia, acute myelogenous leukemia) [77]. Although one-third of the patients enrolled in the phase two clinical trial of HSA-I-131-MIBG had been previously treated with chemotherapy [22], none of these patients developed permanent bone marrow failure after treatment [60]. Most of these patients, however, continue under long-term follow-up with periodic safety evaluations [62]. There have been no studies combining simultaneous CVD and radiolabeled MIBG. A pilot study that combined carboplatin, etoposide, and melphalan (myeloablative chemotherapy) and LSA-I-131-MIBG simultaneously in patients with neuroblastoma indicated that the combination was feasible [79]. Nevertheless, these patients required hematopoietic stem cell rescue. The combination of CVD and radiolabeled MIBG could be evaluated in a clinical trial with a design that includes the option of supportive therapy with autologous stem cell transplant. Patients with MIBG-avid-MPPGs and patients with mixed tumors (MIBG-avid and non-MIBG-avid lesions) may benefit from a trial that combines both therapeutic modalities.

Some chemotherapeutic agents, such as capecitabine and platinum drugs, might sensitize MPPG cells to the lethal radiation emitted by the radiolabeled MIBG [80,81]. Furthermore, a few preclinical studies showed that histone deacetylase inhibitors and hydroxytyrosol increase the expression of the NET in mouse pheochromocytoma cells [82,83]. Although these results are interesting, these agents need to be tested in clinical trials together with radiolabeled MIBG. Other preclinical studies with arsenic indicated that this element could sensitize neuroblastoma cells to radiolabeled MIBG [84]. However, a phase two clinical trial combining LSA-I-131-MIBG and arsenic in patients with neuroblastomas and MPPGs showed that the addition of arsenic did not improve response rates compared with LSA-I-131-MIBG alone [85].

### 8.3. Tyrosine Kinase Inhibitors and I-131-MIBG

Molecular profiling of MPPG has led to the identification of pseudohypoxia and angiogenesis as important hallmarks for MPPG development. Cabozantinib and sunitinib are currently being evaluated in phase two clinical trials for patients with MPPG, and preliminary results with cabozantinib indicate that angiogenesis is a very important, druggable tumor driver [30,86]. Therefore, combinations of tyrosine kinase inhibitors and I-131-MIBG could be explored in clinical trials. In fact, a recent publication described a MPPG patient treated with sunitinib and LSA-I-131-MIBG who had a CR with a remission that lasted for nine months [87]. A reduction of the tumor burden with medications such as cabozantinib or sunitinib may increase the efficacy of I-131-MIBG [87]. Furthermore, patients with mixed tumors may benefit from combining these therapies. The trial design would require careful selection of the tyrosine kinase inhibitor doses and a tight blood pressure control to minimize the risk of cardiovascular events [88,89].

It is not clear whether the inhibition of hypoxia-inducible factor 2 alpha could cause antineoplastic effects in patients with MPPGs [86]. A pilot study with PT2977, a hypoxia-inducible factor 2 alpha inhibitor, will explore this issue. If results of the trial indicate antitumor activity, a combination of PT2977 with I-131-MIBG might be attempted, keeping in mind that patients may be at a high risk of anemia.

### 8.4. Other Radiopharmaceutical Agents and I-131-MIBG

The recent approval of lutetium-177-dotatate for the treatment of patients with gastroenteropancreatic neuroendocrine tumors that express somatostatin receptor 2 has raised the interest in this therapy for patients with MPPGs [90], as PPGs frequently express somatostatin receptor 2. The effects of lutetium 177 dotatate in patients with MPPG are currently being evaluated in clinical trials (www.clinicaltrials.gov). If the drug demonstrates antitumor activity, a combination of I-131-MIBG and lutetium 177 dotatate might be explored. A combination of these two radiopharmaceutical agents may yield added efficacy and decreased toxicity, as their mechanisms of action and biodistribution are different. The trial design would require careful dosage selection and consideration for supportive measures such as autologous stem cell transplant, as both agents emit radiation that increase the risk for bone marrow insufficiency and secondary malignancies.

### 8.5. Immunotherapy and I-131-MIBG

MPPG may escape recognition by the immune system via mechanisms linked with hallmarks such as inflammation, tumor necrosis, and pseudohypoxia. There are ongoing clinical trials of immunotherapy for patients with MPPG (www.clinicaltrials.gov) [86]. Several clinical trials have noted that the recognition of some cancers by the immune system is enhanced by prior exposure to lethal radiation [91]. Patients with MPPGs frequently have bone metastases that are palliated with radiation therapy [88]. Although these patients may benefit from immunotherapy, such benefits would likely be localized rather than generalized. In contrast, I-131-MIBG may enhance a systemic response to immunotherapy.

### 8.6. Retreatment with I-131-MIBG

If a patient has positive outcome following treatment with two intermediate doses of I-131-MIBG, perhaps retreatment could be considered. The patient would need to demonstrate adequate baseline laboratory values and have a history of acceptable prior tolerance of the treatment. Any consideration of retreatment would require review of prior estimated dose delivery to normal organs and tissues and repeat dosimetric evaluation.

### 8.7. Other Radiopharmaceuticals that Target the NET

Astatine-211 is a halogen isotope that possesses similar chemical characteristics to iodine-131. Astatine-211 can substitute Iodine-131 at the meta position of MIBG, creating astatine-211-labeled meta-astatobenzylguanidine (At-211-MABG) [92,93,94]. Unlike iodine-labeled MIBG, At-211-MABG decays by emitting alpha particles. Alpha particles emit high levels of radiation that target tissues over a few cell diameters [92]. This medication may therefore be associated with strong antineoplastic effects and minimal toxicity, as normal tissues may get minimal or no exposure to radiation [92]. Like iodine-labeled MIBG, At-211-MABG targets the NET [92]. The use of At-211-MABG in a PC12 mouse model caused a substantial reduction of pheochromocytomas with mild detrimental effects on bone marrow cells [95]. At-211-MABG may therefore be a potential treatment for patients with MPPG [95]. However, preclinical comparative studies with HSA-I-131-MIBG followed by phase one pharmacokinetics and toxicity studies of At-211-MABG are required.

### 8.8. Other Indications for I-131-MIBG

Approximately 90% of neuroblastomas, 70% of gastroenteropancreatic neuroendocrine tumors, and 30% of medullary thyroid carcinomas express the NET [96,97]. In patients with neuroblastoma, positive responses have been noted in approximately 60% of those treated with LSA-I-131-MIBG [98,99,100]. The experience with neuroblastoma has led to new approaches designed to improve the efficacy of iodine-labeled MIBG. These approaches include dose escalation, incorporating treatment within myeloablative regimens (as previously discussed), and multimodality treatment combining iodine-labeled MIBG with chemotherapy [79].

## 9. Conclusions

The historical development of radiolabeled MIBG as a targeted therapy for patients with MPPG has faced multiple setbacks. Nevertheless, the clinical observations derived from patients treated with LSA-I-131-MIBG led to the recognition of modifiable manufacturing aspects to improve the effectiveness of this therapy. The refined product of these efforts, the HSA-I-131-MIBG, is now associated with remarkable clinical benefits in many patients with MIBG-avid MPPGs. The approval of HSA-I-131-MIBG by the FDA has made it possible for patients with MPPGs to benefit from an effective systemic therapy. Furthermore, this approval has opened up a world of therapeutic research opportunities. These opportunities include the potential recognition of molecular and clinical phenotypes that predict a positive response, the identification of effective therapeutic combinations that target multiple hallmarks for MPPG development, the evaluation of doses and schedules to enhance effectiveness and decrease toxicity, and the discovery of mechanisms responsible for long-term antineoplastic effects and longer survival. A brighter future for patients with MPPGs is ahead.

## Figures and Tables

**Table 1:** Differences between LSA- and HSA-I-131-MIBG.

Characteristics	LSA-I-131-MIBG	HSA-I-131 MIBG
Manufacturing process	Simple isotope exchange methodology [47]	Solid phase precursor Ultratrace process [46]
Unlabeled MIBG in each dose	Large amount [46]	None [46]
Chemical mass of unlabeled amount of MIBG in a 500 mCi dose	~12 mg [46]	~0.2 mg [46]
Specific activity of final drug product	~1.59 MBq/μg (low) [44,46]	~92.5 MBq/μg (very high) [46]
Potential efficacy	Low levels of radioactivity delivered to tumor per dose [46]	High levels of radioactivity delivered to tumor per dose [46]
Potential safety	Excess cold MIBG and increased risk for cardiovascular issues [46]	No cold MIBG, low cardiovascular risk [46]
